# Does Irrigation with Treated and Untreated Wastewater Increase Antimicrobial Resistance in Soil and Water: A Systematic Review

**DOI:** 10.3390/ijerph182111046

**Published:** 2021-10-21

**Authors:** Stacy Slobodiuk, Caitlin Niven, Greer Arthur, Siddhartha Thakur, Ayse Ercumen

**Affiliations:** 1 Department of Forestry and Environmental Resources, North Carolina State University, Raleigh, NC 27695, USA; cgniven@ncsu.edu (C.N.); aercume@ncsu.edu (A.E.); 2 Department of Population Health and Pathobiology, North Carolina State University, Raleigh, NC 27695, USA; gkarthur@ncsu.edu (G.A.); sthakur@ncsu.edu (S.T.)

**Keywords:** antimicrobial resistance, resistance genes, resistant bacteria, persistent organic pollutants, wastewater irrigation, wastewater reuse, water scarcity, water insecurity, untreated municipal wastewater, treated municipal wastewater

## Abstract

Population growth and water scarcity necessitate alternative agriculture practices, such as reusing wastewater for irrigation. Domestic wastewater has been used for irrigation for centuries in many historically low-income and arid countries and is becoming more widely used by high-income countries to augment water resources in an increasingly dry climate. Wastewater treatment processes are not fully effective in removing all contaminants, such as antimicrobial resistant bacteria (ARB) and antimicrobial resistance genes (ARGs). Literature reviews on the impact of wastewater irrigation on antimicrobial resistance (AMR) in the environment have been inconclusive and mostly focused on treated wastewater. We conducted the first systematic review to assess the impact of irrigation with both treated or untreated domestic wastewater on ARB and ARGs in soil and adjacent water bodies. We screened titles/abstracts of 3002 articles, out of which 41 were screened in full text and 26 were included in this review. Of these, thirteen investigated irrigation with untreated wastewater, and nine found a positive association with ARB/ARGs in soil. Out of thirteen studies focused on treated wastewater, six found a positive association with ARB/ARGs while six found mixed/negative associations. Our findings demonstrate that irrigation with untreated wastewater increases AMR in soil and call for precautionary action by field workers, their families, and consumers when untreated wastewater is used to irrigate crops. The effect of irrigation with treated wastewater was more variable among the studies included in our review, highlighting the need to better understand to what extent AMR is disseminated through this practice. Future research should assess factors that modify the effect of wastewater irrigation on AMR in soil, such as the degree and type of wastewater treatment, and the duration and intensity of irrigation, to inform guidelines on the reuse of wastewater for irrigation.

## 1. Introduction

Consequences of an ever-growing global population, such as water pollution, climate change, and unevenly distributed water resources, have led to limitations in accessing clean freshwater, driving the need for the reuse and recycling of water resources. Agriculture is the largest user of freshwater and accounts for almost 75% of water use [1]. With the world’s population estimated to reach 10 billion within the next 30 years, agricultural production is predicted to increase by 70%, putting further strain on freshwater resources [2]. Almost 50% of the world’s population uses polluted water sources for agricultural irrigation, and 20 million hectares are estimated to be irrigated with wastewaters [3]. Wastewater has been used in agriculture for centuries in many cities around the world that have a historically low accumulation of rainwater. It is also an increasingly critical alternative source of water in countries that are most impacted by water scarcity, especially those which rely on agriculture for income. For many low-income countries, reusing untreated wastewater is one of the few affordable alternatives to the advanced processes that occur in most wastewater treatment plants in high-income countries [4]; however, increasing stress on water resources has also led high-income countries to reuse domestic wastewater. For example, the U.S. reuses 4% of its treated wastewater, and some states rely on treated wastewater extensively, such as California and Florida, which use approximately half of their treated wastewater for agriculture [5]. China uses reclaimed wastewater for multiple applications, with one-third of its reclaimed wastewater going towards agricultural irrigation [6]. Irrigating crops with wastewater can also be beneficial as it supplies nutrients to the soil, reducing the need for farmers to purchase fertilizer [6]. In addition to agriculture, irrigation with treated wastewater is also used for landscaping and urban parks.

**Figure 1:**
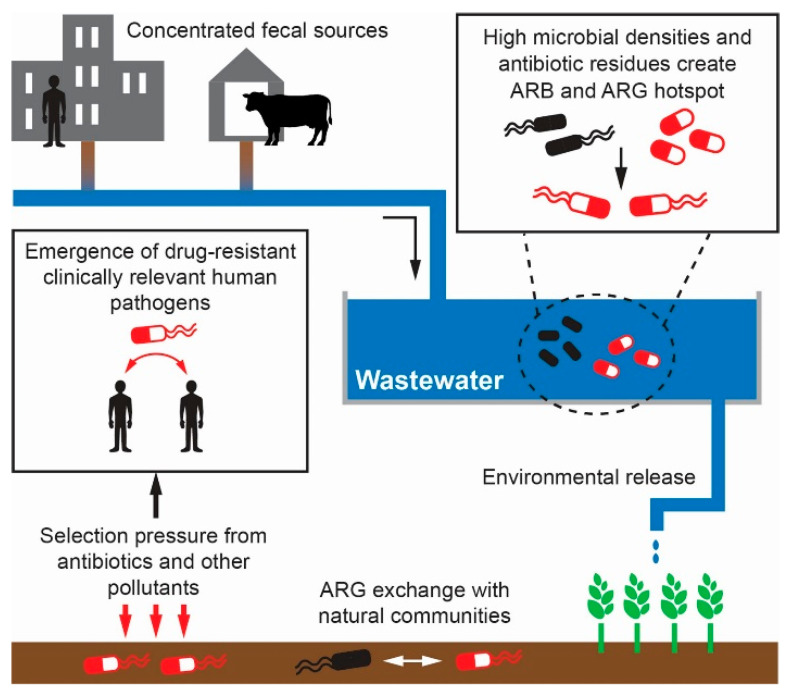
Role of wastewater irrigation in the emergence and spread of antimicrobial resistant bacteria (ARB) and antimicrobial resistance genes (ARGs) in the environment.

Wastewater can also contain high concentrations of heavy metals, pathogens, pharmaceuticals, plastic additives, and other contaminants. Contaminants can adversely affect plant growth when wastewater is applied to crops [7]. Human exposure to wastewater contaminants can also be harmful, and agricultural reuse of wastewater has been associated with health risks. Exposure to wastewater through agricultural irrigation has been linked to enteric diseases such as salmonellosis, shigellosis, cholera, giardiasis, amoebiasis, hepatitis A infections, and viral enteritis among farmers, their families, those living close to wastewater irrigation areas, and consumers of crops irrigated with wastewater [8]. Farmers working in fields that use untreated wastewater for irrigation have also reported experiencing skin irritation, rashes, and dermatitis [8]. Adequate treatment of wastewater prior to agricultural application can alleviate some of these health concerns. However, wastewater treatment processes are not fully effective in removing all contaminants. Contaminants of particular concern include pharmaceuticals, personal care products and antibiotic residues, as well as antimicrobial resistant bacteria (ARB) and antimicrobial resistance genes (ARGs) [9]. Antibiotics are detected in treated wastewater effluent [10] and ARB/ARGs can withstand or even proliferate at treatment plants [11]. Wastewater irrigation can lead to continuous exposure of the irrigated fields to a variety of antibiotics, which can prompt the emergence of resistant strains (Figure 1). ARB in wastewater deposited onto soils by irrigation can also elicit the transfer of ARGs between wastewater bacteria and native soil communities [12]. Crops planted in soil irrigated with wastewater can take up ARB/ARGs [13] and pose a risk of spreading AMR to consumers [14,15]. There is also the potential of ARB/ARG contamination in water bodies that are adjacent to wastewater-irrigated soils [16].

Antibiotics are considered persistent organic pollutants of emerging concern due to their known lasting effects on aquatic environments [17]. Effects of antibiotics on other environmental media are not as well known. Previous non-systematic reviews have provided mixed results on how wastewater irrigation affects AMR in soil. One review concluded that soils irrigated with treated wastewater do not demonstrate an increase in ARB and ARGs [11]. Two other reviews had inconclusive results [12,18]. These reviews were mostly focused on irrigation with treated wastewater and included few studies on irrigation with untreated wastewater. We conducted a systematic literature review to assess the effect of irrigation with both treated and untreated wastewater on the prevalence and abundance of ARB and ARGs in soils and adjacent water bodies. While wastewater from animal sources is often also used for irrigation, and both manure and municipal fecal sludge are used for soil amendment, we focused our review on irrigation with municipal domestic wastewater, either alone or combined with other waste streams.

## 2. Materials and Methods

### 2.1. Search Strategy

We searched the PubMed, Web of Science, CAB Direct, and Agricultural and Environmental Science databases and conducted a search for gray literature in Science.gov. We developed search terms to denote treated and untreated wastewater (e.g., wastewater, sewage, effluent, reclaimed wastewater), agricultural processes that use wastewater (irrigation, agriculture), outcomes of interest (antimicrobial/antibiotic resistance), and environmental reservoirs of interest (e.g., soil, field, surface water, groundwater; Table S1). More detailed information on the PubMed search string can be found in the supplementary information (Text S1). The search was conducted in November 2020. References identified in the database search were imported into Covidence software, where duplicates were removed. Titles and abstracts of the articles were screened using our inclusion and exclusion criteria. For any review articles identified during the title/abstract screening, we screened the bibliographies to identify additional relevant studies. Articles short-listed during the title/abstract screening were reviewed in full text to determine eligibility.

### 2.2. Inclusion and Exclusion Criteria

We included studies if they detected or quantified ARB or ARGs in soils irrigated with wastewater or in water bodies adjacent to wastewater-irrigated soils. Studies were included if any form of treated or untreated domestic wastewater (alone, mixed with industrial/other waste streams, or diluted in an ambient water body after discharge) was used for irrigation. We excluded (1) studies that focused on application or amendment with sewage sludge or biosolids and with solely non-human waste (agriculture waste, dairy waste, piggery waste, manure), (2) studies that covered AMR in aquaculture and marine environments, and (3) experimental lab studies or studies conducted with irrigation water artificially spiked with antibiotics and/or bacteria that would be found in wastewater.

### 2.3. Data Extraction and Synthesis

We extracted relevant data from eligible full-text articles using Microsoft Excel. Extraction was conducted by two reviewers (SS, CN) independently to ensure accuracy, and any discrepancies were resolved by discussion. We reported data on study location, study design, type and treatment of wastewater, duration of irrigation, type of samples tested, analytical methods, prevalence and abundance of ARB and ARGs detected, and additional relevant environmental factors (e.g., pH, soil moisture, seasonality). We qualitatively synthesized data from all eligible articles to separately assess the effect of irrigation with treated and untreated municipal wastewater on the prevalence and abundance of specific ARB/ARGs in soil and adjacent water bodies. We also investigated the effect of additional environmental variables on AMR in soil and water. Our PRISMA checklist can be found in the supplementary information (Table S2).

## 3. Results

**Figure 2:**
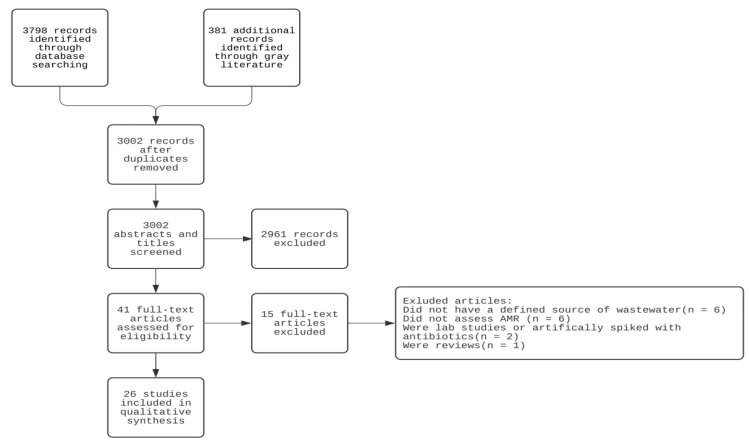
Flowchart of literature review and screening.

We screened the titles/abstracts of 3002 studies and reviewed the full texts of 41 studies. Based on our inclusion/exclusion criteria, a total of 26 studies were eligible to be included in the review (Figure 2). The eligible studies were conducted between 2003 to 2020. Eight studies were conducted in high-income countries (Israel, Spain, Australia, Germany, US) while eighteen studies were based in low-and middle-income countries (Nigeria, Cameroon, Burkina Faso, Egypt, India, Mexico, China). Most studies focused on agricultural fields while four studies investigated urban parks and one study focused on a recharge basin. Twenty-five studies focused on soil samples and only one study investigated subsoil pore water samples.

Thirteen studies focused on irrigation with untreated municipal wastewater and another thirteen studies focused on treated municipal wastewater. The studies included irrigation both directly with wastewater and indirectly using water from an ambient water body that receives wastewater or treated wastewater effluent. Soils irrigated with freshwater, rainfed fields, and pristine soils from remote areas (e.g., national parks) were used as a comparison group. The duration of wastewater irrigation ranged from 1.5 years [18] to 100 years [19,20,21]. Studies used a mix of culture-based and molecular methods and reported the prevalence of antimicrobial-resistant isolates and both absolute abundances of ARGs and relative abundances normalized by 16S rRNA gene counts. The most commonly detected ARB included fecal bacteria and native soil bacteria, such as *Escherichia coli*, *Enterococcus*, *Azotobacter chroococcum*, *Pseudomonas* spp., and *Flavobacteria*. Studies assessed resistance to a wide range of antibiotics, including tetracycline, ampicillin, and ciprofloxacin. Commonly investigated ARGs included tetracycline resistance genes, sulfonamide resistance genes, quinolone resistance genes, and beta-lactamase genes. Some studies focused on detecting genetic elements involved in horizontal gene transfer, such as class 1 integrons (*intl1*).

### 3.1. Irrigation with Untreated Wastewater

Out of thirteen studies focused on untreated wastewater, one studied solely domestic wastewater, seven studied wastewater that was a combination of domestic and industrial, hospital, agriculture, market or slaughterhouse waste, and five referred to municipal wastewater without specifying the content (Table 1). Three studies had no comparison group to allow assessment of associations but detected ARB in soils irrigated with untreated wastewater [22,23,24]. Of the ten studies with a comparison group, nine found that wastewater irrigation was associated with increased ARB/ARGs in soil (Table 1). In one of these nine studies, the wastewater came indirectly from a waterbody [25]. Additional details of the studies are provided in the Supplemental Information (Table S3).

Four studies were conducted in Mezquital Valley in Mexico, one of the world’s largest wastewater irrigation systems, where untreated wastewater from Mexico City has been used to irrigate farmlands for 100 years [26]. All of these studies found a positive association between wastewater irrigation and ARB/ARGs in soil. Studies at this site also took advantage of the long history of wastewater irrigation to assess whether ARB/ARGs in soil increase with increasing duration of irrigation. One study found substantially more isolates resistant to at least one antibiotic in wastewater-irrigated fields (51%) than in rainfed fields (6%) and a higher prevalence (25%) of isolates resistant to ≥2 antibiotics in wastewater-irrigated fields than in rainfed fields (6%) [20]. Another study found the absolute abundance of *sul1* genes to be 150–1500 times higher and *sul2* genes 50–520 times higher in wastewater-irrigated soils than in rainfed soils; the relative abundance of both genes was also higher in wastewater-irrigated soils. While the absolute abundance of both genes increased with increasing years of irrigation, the relative abundance did not; soils irrigated for 100 years did not contain more *sul1* and *sul2* genes on the relative scale compared to soils irrigated with wastewater for 1.5 years [19]. A similar study at this site showed significant positive correlations between absolute gene abundance and years of irrigation for *intl1*, *korB*, *tetW*, *aadA*, and *qacE* + *qacE*Δ1 (quaternary ammonium compound resistance) genes while the relative abundance of these genes did not vary with duration of wastewater irrigation [21]. A fourth study from Mezquital Valley compared a field that has been irrigated with untreated wastewater for over 80 years to a rainfed field that had never been irrigated. Soil samples from the wastewater-irrigated field had an absolute abundance of 3.3 × 10^6^ gene copies of *sul1* genes per g of soil compared to 3.1 × 10^5^ gene copies per g in samples from the rainfed field while *sul2* genes were only detected in the wastewater-irrigated field [27]. In a further experiment in the same study, where soil cores from both fields were irrigated with wastewater with and without sulfamethoxazole and ciprofloxacin, the relative abundance of *sul1* genes in soil from the rainfed field increased by up to 3 orders of magnitude after the irrigation experiment, while it increased by <1 order of magnitude in soil from the wastewater-irrigated field.

Other investigations of irrigation with untreated wastewater included an additional study in Mexico and studies conducted in Egypt, China, Cameroon, Burkina Faso, and India. In Mexico, water from a river that receives discharges of untreated domestic wastewater from the city of Chihuahua was used to irrigate two agricultural fields. Irrigation with wastewater-impacted river water stopped on one of the fields 14 years prior to the study but continued on the other. The field continuing to receive wastewater-impacted river water showed a higher number of multidrug-resistant bacteria compared to both the field that no longer receives water from the river and a control field that was rainfed [25]. In a study in Egypt, the incidence of plasmids was 25–50% higher in isolates from wastewater-irrigated soil than from soils irrigated with canal water, and >50% of isolates carrying plasmids were resistant to ampicillin and kanamycin while >25% were resistant to tetracycline [28]. A study in China compared agricultural fields, one irrigated with untreated domestic wastewater for over twenty years and a second irrigated with fishpond water, to a field that was not used for cultivation. While the soils irrigated with fishpond water had higher *tet* and *sul* relative gene abundances than the wastewater-irrigated fields, ARGs were not detected in the field not used for cultivation [29]. In Cameroon and Burkina Faso, a study researched the impact of irrigation with raw sewage receiving input from homes, hospitals, agriculture, markets, and slaughterhouses compared to non-irrigated soils. Transferable ARGs conferring resistance to trimethoprim, aminoglycosides, beta-lactams, amphenicols, tetracyclines, sulfonamides, macrolides, quinolones, phosphonic antibiotics, and nucleoside antibiotics were 27% more abundant in wastewater-irrigated soils than in non-irrigated control soils [3]. An additional publication from the same study investigated different AMR mechanisms in both fields, including the presence of genes encoding antibiotic inactivation enzymes, antibiotic target replacement, antibiotic target protection and efflux pumps. The study found the number of ARGs encoding antibiotic inactivation enzymes to be lower in the non-irrigated fields compared to the wastewater-irrigated fields, and the number of ARGs encoding other resistance mechanisms were slightly higher in wastewater-irrigated fields [30].

In four studies in India, fields were irrigated for at least a decade with wastewater that came from factories and domestic sewage. When compared to a groundwater-irrigated field, *Pseudomonas* spp. isolated from the wastewater-irrigated field had higher resistance towards sulphadiazine, ampicillin, and erythromycin [31]. The other three studies investigated wastewater-irrigated fields in the same area but did not report results from a comparison field. All three studies detected various ARB in wastewater-irrigated fields. These included free-living *Azotobacter chroococcum* isolates resistant to nitrofurantoin (92%), polymyxin-B (86%), co-trimoxazole (81%) and a total of six antibiotics (41%) [22], bacterial isolates resistant to tetracycline (75%), doxycycline (58%), ampicillin (50%), and nalidixic acid (50%) [23], and *Pseudomonas* spp. isolates resistant to cloxacillian (100%), methicillin (58%) and a total of four antibiotics (25%) [24].

### 3.2. Irrigation with Treated Wastewater

Out of thirteen studies focused on treated wastewater, the wastewater effluent was secondary-treated in three studies, a mix of secondary- and tertiary-treated in one study, tertiary-treated in three studies and biologically treated with a wetland system in one study (Table 2). The remaining five studies did not report the extent of treatment. In three studies, the wastewater effluent was diluted prior to irrigation by discharging into an ambient waterbody. Eight studies focused on agricultural fields, four on urban parks and one on a water storage basin recharged with treated wastewater. One study investigated pore water samples while the rest investigated soil. One study did not utilize a comparison field for assessing associations but found ARB in wastewater-irrigated fields. Of the twelve studies that had a comparison site, six found that wastewater irrigation was associated with higher ARB/ARGs in soil while four studies found mixed associations, and in two studies, wastewater irrigation was associated with lower or similar ARB/ARGs in soil compared to irrigation with freshwater, groundwater, and non-irrigated fields. Additional details of the studies are provided in the Supplemental Information (Table S3).

The six studies that found a positive association were conducted in China, Australia, Mexico and Germany. In China, a study compared a field irrigated with treated wastewater (either directly or indirectly from a river receiving discharge), a field that was irrigated with untreated wastewater until 6–7 years ago and with rain- and groundwater since then, and a third field that is non-irrigated. The study found that the relative abundance of sulfadiazine-resistant bacteria was highest in the field previously irrigated with untreated wastewater, with no other differences in the relative abundance of ARBs between the fields. The relative abundance of *tetA*, *tetC*, *tetE*, *tetG*, *tetS*, *sul1* and *sul3* genes as well as the sum of the relative abundances of *tet* and *sul* genes were significantly higher in currently and previously wastewater-irrigated soils than in non-irrigated soils [32]. There was no difference in relative abundance of ARGs between currently and previously wastewater-irrigated soils. Two other studies in China focused on public parks irrigated with reclaimed wastewater but did not report the type and degree of treatment. In one of these studies, there was a higher diversity and abundance of ARGs encoding beta-lactam, FCA (fluoroquinolone, quinolone, florfenicol, chloramphenicol, amphenicol), and aminoglycoside resistance in soil from urban parks irrigated with reclaimed wastewater than in control soils from urban parks in the same cities not irrigated with wastewater [33]. The abundance of ARGs was 99–8655 times higher in wastewater-irrigated parks while the abundance of transposase genes was up to 2959 times higher compared to control soils. The second study also found higher diversity and absolute abundance of *sul1* genes (1.69 × 10^8^ copies per g dry soil) and *intl1* genes (7.62 × 10^7^ copies per g dry soil) in soil irrigated with reclaimed water than in pristine soils from national parks (9.08 × 10^7^ and 2.61 × 10^7^ copies per g dry soil) [34].

A similar study in Australia compared urban parks irrigated with tertiary-treated wastewater, urban parks irrigated with potable water and remote national parks. Wastewater-irrigated parks had a higher number of different ARGs than both other sites. The abundance of ARGs in soil from wastewater-irrigated parks, conferring resistance to all major classes of antibiotics, except for erythromycin and vancomycin, was 815–4300 times higher than soil from a national park. Urban parks without wastewater irrigation, on the other hand, had 150–1240 times higher prevalence of ARGs than soil from the national park [35]. There was no difference in the relative abundance of *intl1* and *tnpA* genes between sites. In a study in Mexico, soil from recreational parks irrigated with tertiary-treated wastewater had a higher number of multi-drug resistant bacteria in parks closer to the wastewater treatment plant compared to parks further away [36].

Finally, a study in Germany compared ARGs in subsoil pore-water in fields irrigated with secondary-treated wastewater during periods of different irrigation intensity and a period with no irrigation. The relative abundance of *sul*, *tet*, *qnr*, *bla* and *intl1* genes was higher during high-intensity irrigation compared to the irrigation break, and the relative abundance of several ARGs increased with increasing irrigation intensity [37]. A lab study was set up to replicate the field study and confirmed that the relative abundance of ARGs was higher in soils irrigated with treated wastewater versus freshwater [37]. Additionally, a study in Nigeria investigated soil irrigated with secondary-treated wastewater. While the study did not use a comparison site, 100% of *E. coli* isolates from wastewater-irrigated soils were resistant to ≥5 antibiotics [38].

The six studies that found mixed or negative associations between wastewater irrigation and ARB/ARGs in soil were conducted in Spain, Israel and the US. Two studies in Spain investigated fields irrigated with wastewater from a channel that received up to 92% effluent from 10 wastewater treatment plants versus fields irrigated with rain- or groundwater. In the first study, the relative abundance of *tetM*, *mec*A, *qnrS1* and *bla*_OXA-58_ genes was higher in wastewater-irrigated fields, but the relative abundance of *bla*_CTX-M-32_ was higher in the groundwater-irrigated areas [39]. The second study also investigated a third field irrigated with wastewater-impacted river water, where wastewater effluent made up <18% of the water flow. The abundance of *intl1* genes was higher in soil irrigated with groundwater but the highest abundance of *bla*_TEM_ was found in the soils irrigated with river water containing <18% wastewater effluent, while the abundance of *qnrS1* genes was higher in both wastewater-irrigated fields [40].

In Israel, a study compared fields irrigated with secondary-treated wastewater to fields irrigated with freshwater, including groundwater from an aquifer recharged with secondary-treated wastewater. The relative abundance of ARB was similar or higher in the freshwater-irrigated soils. Absolute gene copy numbers for ARGs tested (*sul1, sul2, ermB, ermF, tetO*, and *qnrA*) were similar or higher in the freshwater-irrigated soils at three out of four study sites while they were higher in wastewater-irrigated soils at the remaining site. Similarly, the relative abundance of ARGs was higher in the freshwater-irrigated soils at three sites and higher in wastewater-irrigated soils at the fourth site [41]. Notably, one of the comparison sites in this study was irrigated with groundwater from an aquifer that is recharged with secondary-treated wastewater. In a second study in Israel, commercial agriculture fields irrigated with secondary- and tertiary-treated wastewater were compared to fields irrigated with surface water, groundwater, or desalinated water. The study also examined an experimental orchard and lysimeters irrigated with tertiary-treated wastewater and freshwater. Wastewater-irrigated soil in lysimeters had higher relative and absolute abundance of *intl1* genes compared to freshwater-irrigated lysimeters. However, almost all ARGs were below detection limits in all tested soils, even after irrigation with treated wastewater [42]. A third study in Israel compared soils irrigated with greywater treated by constructed wetlands to soils irrigated with freshwater, with no difference in the abundance of tetracycline-resistant bacteria between the two types of soils [43].

Finally, a study in the U.S. investigated *Enterococcus* from sediments of a basin recharged with tertiary-treated wastewater for more than 20 years and compared it to enterococci isolated from soils and sediments in a groundwater-filled pond. A higher proportion of bacteria isolated from the groundwater-filled pond was resistant to 4–6 antibiotics (25%) than bacteria from the wastewater-recharged pond (9%), and a smaller proportion of bacteria from the groundwater-filled pond was susceptible to all antibiotics tested (7%) than bacteria from the wastewater-recharged pond (36%) [44].

### 3.3. Effect of Other Environmental Factors

Other environmental factors besides wastewater irrigation had impacts on the abundance and diversity of AMR in soil. Multiple studies noted the impact of soil moisture, precipitation, temperature, pH and soil depth. A study in Israel found soil moisture had a significant positive correlation with bacterial resistance to tetracycline and ciprofloxacin [41]. A study in Germany found that the relative abundance of *sul1* and plasmid-borne *qnrS* genes in subsoil pore water increased with increasing temperature, and the relative abundance of *sul1* genes was positively correlated with precipitation, but there was no correlation between ARGs and humidity [37]. Similarly, in Mexico, the prevalence of ARB in wastewater-irrigated soils was lower during the dry sampling period compared to the rainy period [36]. Evidence on the effect of soil pH was mixed. A study in Australia found ARG abundance in soil to increase with soil pH [35] while conversely, in another study, higher soil pH was negatively correlated with the abundance of ARGs and the *intl1* gene [34]. In a study in Mexico, there was no association between soil pH and ARG abundance [21]. Most studies investigated top soils (0–30 cm depth) and some studies assessed the effect of soil depth on ARB/ARGs. In Mexico, the prevalence of multi-resistant bacteria was not affected by soil depth, comparing samples collected at 0–15, 15–30 and 30–50 cm depth [25]. Similarly, In China, the relative abundance of ARGs was not significantly different between soil depths of 0–10 cm and 10–20 cm [29]. Aggregation of agricultural soil may also play a role in the dissemination of AMR in wastewater-irrigated fields. A study in China found no difference in ARG abundance between rhizosphere, non-rhizosphere and wetland samples [34]. In a study in Mexico, untreated domestic wastewater used to irrigate soil cores was dyed before irrigation to visualize water flow paths. The dye stained a greater volume and deeper in the soil cores collected from wastewater-irrigated fields (80%) than those in the more compacted rainfed fields where the dyed water followed the root system rather than penetrating a larger area of the soil core (50%). The abundance of *sul1* and *sul2* genes was higher in stained soil compartments along the flow path than in unstained compartments, suggesting that water flow paths could be an area of concern with high levels of resistance genes [27].

## 4. Discussion

This review summarizes results from 26 studies on the impact of wastewater irrigation on the prevalence and abundance of ARB and ARGs in soil and water. Our review indicates that an important determinant of the presence of AMR in wastewater-irrigated soil is whether the wastewater used for irrigation was treated. We found evidence of a positive relationship between irrigation with untreated wastewater and both the presence and abundance of ARB/ARGs in soil, where nine out of ten studies that had a comparison group (e.g., fields irrigated with freshwater) showed an increase in ARB and ARGs in wastewater-irrigated soils. In contrast, studies that investigated irrigation with treated wastewater had heterogeneous findings. Out of the twelve studies in this category that had a comparison group, wastewater irrigation was associated with more abundant ARB/ARGs in soil in six studies, while the remaining six studies found mixed or negative associations.

Our review also revealed that studies examining ARB and ARGs in water bodies due to wastewater irrigation are currently limited. Only one study in our review studied sub-pore water, and we identified no studies investigating AMR in underlying groundwater aquifers or surface water bodies adjacent to wastewater-irrigated fields. Wastewater irrigation has been associated with the detection of pathogens, nitrates, and antibiotics in surface- and groundwaters [45]. Future research should investigate whether ARB/ARGs are detected in waters impacted by wastewater irrigation.

### 4.1. Differences in Wastewater Treatment

Our findings highlight the need to further investigate the drivers of heterogeneity to identify settings and factors that modify the risk associated with wastewater irrigation. Notably, the studies focused on untreated wastewater exclusively came from middle and low-income countries while eight out of thirteen studies on treated wastewater came from high-income countries. AMR carriage is significantly higher in low-income countries, which has been attributed to unregulated antibiotic use and poor sanitary conditions [46,47]. Therefore, wastewater used for irrigation in low-income countries is more likely to contain ARB/ARGs. The extent and effectiveness of wastewater treatment also differs between high- and low-income countries. The six studies that investigated irrigation with treated wastewater and found mixed or negative effects on ARB/ARGs in soil were conducted in high-income countries with presumably effective and well-operating wastewater treatment systems, while the majority of the studies that found an increase in ARB/ARGs in soil from irrigation with treated wastewater came from low-income countries. Therefore, differences in ARB/ARG loads in wastewater and removal efficiency for ARB/ARGs in wastewater treatment plants between high- and low-income countries could explain why studies on irrigation with untreated wastewater found an increase in AMR in soil while studies on irrigation with treated wastewater had heterogeneous findings.

Differences in the types of wastewater treatment steps employed would also be expected to affect the presence of antibiotics, ARB, and ARGs in the treated effluent and consequently the impact on soils. However, studies in our review that focused on secondary vs. tertiary-treated wastewater had similarly mixed findings. Among the three studies that investigated tertiary-treated wastewater, two found a positive association between wastewater irrigation and ARB [36] and ARGs [35] while the third found a negative association with ARB [44]. Whether or not the wastewater or treated effluent was diluted via discharge into an ambient waterbody prior to irrigation also did not appear to influence the effect of wastewater irrigation on ARB/ARGs in soil.

### 4.2. Duration of Irrigation

It is possible that ARB from irrigation with wastewater could take several years to accumulate in soil [48]. The duration of wastewater irrigation prior to sampling varied immensely (1.5 to 100 years) across the studies in our review, and nine out of 26 studies did not report the duration. Based on studies in Mezquital Valley, duration of irrigation has implications for the dissemination of ARB/ARGs within wastewater-irrigated soils [19,21]. Therefore, mixed findings between studies could be due to differences in the duration of wastewater irrigation. In addition, when ARB and ARGs are detected in wastewater-irrigated soils, it is unknown whether and how long they persist, either through the survival of the host bacteria or as free naked DNA [18]. Few studies in our review reported the time elapsed between the last episode of wastewater irrigation and collection of samples, and in most studies, soils appeared to be sampled concurrently with ongoing wastewater irrigation. Three studies investigated fields where wastewater irrigation was discontinued and had mixed findings. In Spain, the field currently irrigated with untreated wastewater had the highest abundance of multi-resistant bacteria while the field previously irrigated with untreated wastewater and the rainfed control field had similar abundance of multi-resistant bacteria [25]. In a study in Germany, the relative abundance of ARGs was higher during periods with active irrigation compared to after a 4-month irrigation break [37]. In contrast, in China, there was no difference in the relative abundance of ARB and ARGs between fields currently vs. previously irrigated with wastewater [32]. Other facets of wastewater irrigation, such as the origin of wastewater, and the intensity, frequency and volume of irrigation can also modify the effect of wastewater irrigation on AMR in soil; these factors were only partially reported by studies in our review.

### 4.3. Pre-Existing AMR in Soil

Mechanisms for AMR naturally exist in native soil communities [49]. When determining the impact of wastewater irrigation on ARB and ARGs, it can be difficult to assess the respective contribution of wastewater due to the natural bacteria and resistance already present in the soil [41]. DNA can exist in soil for long periods of time so when researchers use molecular methods of detection, such as polymerase chain reaction (PCR), they might detect pre-existing native bacteria that have been in the soil for many years [12]. Many studies in our review included soil samples that were not wastewater-irrigated, allowing a comparison to isolate the impact of wastewater. However, comparison soils can also be contaminated with AMR elements if they are close to wastewater-irrigated sites (via aerosols and dust) or if they have received soil amendment with manure or biosolids [19]. Only a few studies in our review used “pristine” comparison soils from remote areas with less anthropogenic activity.

### 4.4. Analytic Methods and Detection Limits

Selection of which ARB/ARGs were investigated can also lead to heterogeneous findings across studies. It is also important to note that due to limits of detection and quantification, PCR can fail to detect or quantify ARGs that are present in low levels, which may still have a biological impact [18]. Levels of ARGs can be expressed as a gene ratio, comparing the gene copy numbers of the ARG to those of a common gene such as 16S rRNA. These ratios are used to define the relative abundance of the ARG and can be too low to be interpreted or compared between samples [18].

### 4.5. Environmental Factors

Finally, it is important to include details of soil properties and environmental characteristics in future studies. Multiple studies in our review indicated associations between the abundance of ARGs and soil pH, soil moisture, precipitation and temperature. Additionally, the fate and transport of antibiotics in soil and the DNA extraction efficiency from soil samples can vary with varying soil properties [41]. Reporting soil and environmental characteristics in future studies could help identify factors that may modify the effect of wastewater irrigation on the presence and abundance of ARB/ARGs in soil.

### 4.6. Potential Human Health Risks

Studies have attempted to estimate the human health risk from exposure to antibiotics through wastewater irrigation [15,50], but the health effects of potential exposure to ARB and/or ARGs due to wastewater irrigation are unclear [18,51]. Individuals can be exposed to these through contact with soil or consumption of crops that have taken up ARB/ARGs from wastewater-irrigated soil, potentially leading to gut colonization with resistant bacteria. However, environment-to-human transmission of AMR remains poorly understood [52]. Studies using advanced molecular techniques such as whole genome sequencing have shown genetic overlap between ARB isolated from humans, animals and the farm environment, suggesting transmission between these reservoirs and hosts through farming practices such as soil amendment with manure [53,54]. Similar risks could exist for farmers as well as consumers due to environment-to-human transmission and spread of AMR when untreated wastewater is used for irrigation [55]. Low-income countries, where most wastewater remains untreated and is also more likely to contain ARB/ARGs due to high community carriage rates, are a particularly high-risk setting for further emergence and spread of AMR via wastewater irrigation. Novel resistance mechanisms that emerge in such hotspots have been shown to quickly spread globally [56,57]. Irrigation with untreated wastewater could therefore pose risks beyond the countries where it is practiced.

## 5. Conclusions

Given scarce water resources, climate change and population growth, wastewater irrigation is increasingly common, in both low- and high-income countries. Through a systematic review and synthesis of the available literature, we demonstrate the diverse impact that domestic wastewater irrigation can have on the presence of AMR in soil. Our findings indicate a clear relationship between untreated wastewater irrigation and increasing prevalence and abundance of ARB and ARGs in soil. While there are no studies on the magnitude of human health risks associated with exposure to AMR via irrigation with untreated wastewater, our findings warrant precautionary action by field workers, their families, and consumers, particularly in low-income countries where use of raw sewage for irrigation is common. Studies should also investigate whether irrigation with untreated wastewater leads to contamination of adjacent water sources with ARB and ARGs. In our review, the evidence on whether irrigation with treated wastewater increases the prevalence and abundance of AMR in soil was mixed. Future research should explore factors that can explain the heterogeneity in the effect of irrigation with treated wastewater on ARB and ARGs in soil, such as the extent of wastewater treatment and the intensity of irrigation, to inform guidelines on wastewater reuse for irrigation.

## Figures and Tables

**Table 1:** Characteristics of studies on irrigation with untreated wastewater.

Author and Year	Location	WWI Site	WWI Duration	Comparison Site	Organism	AMR Mechanism Investigated	Reference
Aleem et al., 2003	India	Field irrigated with untreated industrial wastewater mixed with domestic sewage	10 years	Field irrigated with groundwater	*Azotobacter chroococcum* isolates	Resistance against: Amoxycillin, Cloxacillin, Co-trimoxazole, Doxycycline, Methicillin, Nitrofurantoin, Polymyxin-B, Rifampicin, Streptomycin, Sulphadiazine, Tetracycline	[22]
Ansari et al., 2007	India	Field irrigated with untreated industrial wastewater mixed with domestic sewage	>20 years	None	Bacterial isolates	Resistance against: Ampicillin, Chloramphenicol, Ciprofloxacin, Co-trimoxazole, Doxycycline, Gentamicin, Kanamycin, Nalidixic acid, Neomycin, Streptomycin, Tetracycline	[23]
Bahig et al., 2008	Egypt	Field irrigated with untreated wastewater	Not reported	Field irrigated with canal water	Bacterial isolates	Resistance against: Ampicillin, Tetracycline, Kanamycin	[28]
Bougnom et al., 2019	Burkina Faso, Cameroon	Field irrigated with untreated domestic wastewater mixed with hospital, agriculture, market and slaughterhouse waste	20 years	Non-irrigated field	N/A ^a^	ARGs encoding: Antibiotic inactivation enzymes, antibiotic target replacement, antibiotic target protection, efflux pumps	[3]
Bougnom et al., 2020	Same as above	Same as above	Same as above	Same as above	Same as above	Non-targeted ARGs and *Enterobacteriaceae* plasmid replicons	[30]
Broszat et al., 2014	Mexico	Field irrigated with untreated municipal wastewater	8, 10, 85, and 100 years	Rain-fed field	Bacterial isolates	Resistance against: Ampicillin, Chloramphenicol, Erythromycin, Gentamicin, Kanamycin, Oxacillin, Streptomycin, Ciprofloxacin, Doxycycline, Tetracycline, Vancomycin, Sulfamethoxazole ARGs: Sulfonamide (*sul*) and fluoroquinolone (*qnr*) resistance genes	[20]
Dalkmann et al., 2012	Mexico	Field irrigated with untreated municipal wastewater	1.5, 3, 6, 8, 85, and 100 years	Rain-fed field	N/A ^a^	ARGs: Sulfonamide resistance genes (*sul1*, *sul2*), fluoroquinolone resistance genes (*qnrA*, *qnrB*, *qnrS*)	[19]
Jechalke et al., 2015	Mexico	Field irrigated with untreated municipal wastewater (65% domestic sewage, 20% service sector waste, 15% industrial waste)	1.5, 3, 6, 8, 85, and 100 years	Rain-fed field	N/A ^a^	ARGs: *tetW, tetQ, aadA, qacE* + *qacE*Δ*1* Mobile genetic elements: *intI1*, IncP-1plasmids (*korB*)	[21]
Lüneberg et al., 2017	Mexico	Field irrigated with untreated wastewater	>80 years	Rain-fed field	N/A^a^	ARGs: *sul1, sul2, qnrB, qnrS*	[27]
Malik and Aleem 2011	India	Field irrigated with untreated industrial wastewater mixed with domestic sewage	10 years	Field irrigated with groundwater	*Pseudomonas* spp. isolates	Resistance against: Amoxycillin, Ampicillin, Chloramphenicol, Ciprofloxacin, Cloxacillin, Cotrimoxazole, Doxycycline, Erythromycin, Gentamicin, Kanamycin, Methicillin, Nalidixic acid, Neomycin, Nitrofurantoin, Polymyxin-B, Rifampicin, Streptomycin, Sulphadiazine, Tetracycline	[31]
Palacios et al., 2017	Mexico	(1) Field irrigated with water from river that receives untreated wastewater (2) Field irrigated with untreated wastewater from river until >10 years ago	Not reported	Rain-fed field	Bacterial isolates	Resistance against: Ampicillin, 24 additional antibiotics (6 for Gram-negative bacteria, 6 for Gram-positive bacteria, 12 for both)	[25]
Pan and Chu 2018	China	(1) Fields irrigated with untreated domestic wastewater (2) Fields irrigated with fishpond water	>20 years	Field with no cultivation	N/A ^a^	ARGs: Tetracycline (*tetA, tetB, tetC, tetE, tetM, tetO, tetS, tetX*) and sulfonamide resistance genes (*sul1, sul2, sul3*)	[29]
Shafiani and Malik 2013	India	Field irrigated with untreated industrial wastewater mixed with domestic sewage	10 years	None	*Pseudomonas* spp. isolates	Resistance against: Amoxycillin, Chloramphenicol, Cloxacillin, Doxycycline, Methicillin, Nalidixic acid, Tetracycline	[24]

WWI: Wastewater irrigation; AMR: Antimicrobial resistance; ARG: Antimicrobial resistance gene. ^a^ No specific target organism, DNA extracted directly from soil.

**Table 2:** Characteristics of studies on irrigation with treated wastewater.

Author and Year	Location	WWI Site	WWI Duration	Comparison Site	Target Organism	AMR Mechanism Investigated	Reference
Cerqueira et al., 2019	Spain	Field irrigated with water from channel with up to 92% treated effluent from 10 wastewater treatment plants	Not reported	Field irrigated with ground- and/or rainwater	N/A ^a^	ARGs: *sul1, bla*_TEM_, *bla*_OXA-58_, *bla*_CTX-M-32_, *mec*A, *qnrS1, tetM* Mobile genetic elements: *intl1*	[39]
Cerqueira et al., 2019	Spain	(1) Field irrigated with water from channel with up to 92% treated effluent from 10 wastewater treatment plants (2) Field irrigated with water from river that contains <18% treated effluent	Not reported	Field irrigated with groundwater	N/A ^a^	ARGs: *sul1, bla*_TEM_, *bla*_OXA-58_, *bla*_CTX-M-32_, *mec*A, *qnrS1, tetM* Mobile genetic elements: *intl1*	[40]
Chen et al., 2014	China	(1) Field irrigated with treated wastewater directly or from rivers that receive effluent (2) Field irrigated with untreated wastewater until 6–7 years ago, irrigated with ground- and/or rainwater since	Not reported	Non-irrigated field	Bacterial isolates DNA from soil	Resistance against: Oxytetracycline, Tetracycline, Sulfadiazine, Sulfamethoxazole ARGs: 13 tetracycline resistance genes (*tetA, tetB, tetC, tetD, tetE, tetG, tetK, tetL, tetM, tetO, tetS, tetQ, tetX*), 3 sulfonamide resistance genes (*sul1, sul2, sul3)*	[32]
Chigor et al., 2020	Nigeria	Earthen pots irrigated with secondary treated wastewater	Practiced in the area for >30 years, earthen pots irrigated for 6 weeks	None	*E. coli* isolates	Resistance against: Amoxicillin, Ampicillin, Penicillin, Cloxacillin, Cefuroxime, Streptomycin, Rifampicin, Metronidazole, Sulfamethoxazole, Trimethoprim, Vancomycin, Erythromycin, Clarithromycin, Chloramphenicol, Ciprofloxacin, Norfloxacin, Tetracycline, Imipenem	[38]
Han et al., 2016	Australia	Urban park irrigated with tertiary treated wastewater	Not reported	(1) Urban park irrigated with potable water (2) Pristine soil from remote national parks	N/A ^a^	84 ARGs encoding resistance to aminoglycosides, Classes A, B, C and D beta-lactam, erythromycin, quinolones and fluoroquinolones, macrolide lincosamide streptogramin_b (MLS_b), multidrug, tetracycline, vancomycin Mobile genetic elements: *intI1, tnpA* gene of IS6 family transposons	[35]
Kampouris et al., 2020	Germany	Field irrigated with secondary treated wastewater, sometimes mixed with digested sludge	50 years	(1) Period of irrigation compared to period without irrigation (2) Lab experiment where soils were irrigated with treated wastewater and freshwater	N/A ^a^	ARGs: *sul1, tetM, qnrS, bla*_OXA-58,_ *bla*_CTX-M-32,_ *bla*_TEM_ Mobile genetic elements: *intI1*	[37]
Marano et al., 2019	Israel	Fields irrigated with secondary and tertiary treated wastewater	Not reported	(1) Field irrigated with surface-, ground- or desalinated water (2) Experimental orchard and lysimeters irrigated with tertiary treated wastewater vs. freshwater	N/A ^a^	ARGs: *bla*_GES._ *bla*_OXA2,_ *bla*_OXA10,_ *bla*_TEM,_ *bla*_CTX-M-32,_ *qnrS* Mobile genetic elements: *intl1*	[42]
McLain and Williams 2010	USA	Soil from water storage basin recharged with tertiary treated wastewater	>20 years	Soil from water storage basin recharged with groundwater	*Enterococcus* isolates	Resistance against: Tigecycline, Tetracycline, Chloramphenicol, Daptomycin, Streptomycin, Tylosin tartrate, Quinupristin/dalfopristin, Linezolid, Nitrofurantoin, Penicillin, Kanamycin, Erithromycin, Ciprofloxacin, Vancomycin, Lincomycin, Gentamicin	[44]
Negreanu et al., 2012	Israel	Fields irrigated with secondary treated wastewater	6, 12, 15 years	Field irrigated with freshwater, including aquifer recharged with secondary treated wastewater	Bacterial isolates DNA from soil	Resistance against: Tetracycline, Ciprofloxacin, Erythromycin ARGs: *sul1, sul2, ermB, ermF, tetO, qnrA*	[41]
Palacios et al., 2017	Mexico	Recreational parks irrigated with tertiary treated wastewater	Not reported	Distance from WWTP	Bacterial isolates	Resistance against: Ampicillin, Riphampicin, Chloramphenicol, Ciprofloxacin, Gentamicin, Trimethoprim-sulphametoxazole	[36]
Troiano et al., 2018	Israel	Field irrigated with greywater treated by recirculating vertical flow constructed wetland	>7 years	Field irrigated with freshwater	Bacterial isolates	Resistance against: Tetracycline, Amoxicillin, Ciprofloxacin, Kanamycin ARGs: Beta-lactamase genes (*bla*_TEM_, *bla*_CTXM-32_, *bla*_SHV_, *bla*_OXA-2_, *bla*_OXA10_), tetracycline resistance genes (*tet39*, *tetA*, *tetB tetM*, *tetQ*, *tetW*)	[43]
Wang et al., 2014	China	Public parks irrigated with treated wastewater	Not reported	Pristine remote parks	N/A ^a^	ARGs: 15 tetracycline resistance genes, 4 beta-lactamase genes, 3 quinolone resistance genes Mobile genetic elements: *intl1*	[34]
Wang et al., 2014	China	Urban parks irrigated with treated wastewater in seven cities	3–12 years	Urban parks not irrigated with reclaimed water in same cities	N/A ^a^	ARGS: 285 different ARGs Mobile genetic elements: 9 transposase genes	[33]

WWI: Wastewater irrigation; AMR: Antimicrobial resistance; ARG: Antimicrobial resistance gene. ^a^ No specific target organism, DNA extracted directly from soil.

## Supplementary Materials

The following are available online at https://www.mdpi.com/article/10.3390/ijerph182111046/s1, Table S1: Search terms, Text S1: PubMed search string, Table S2: PRISMA checklist, Table S3: Characteristics of studies included in review.
