# Supplementary material for: Does Irrigation with Treated and Untreated Wastewater Increase Antimicrobial Resistance in Soil and Water: A Systematic Review
Source: Int J Environ Res Public Health. 2021 Oct 21;18(21):11046. doi: 10.3390/ijerph182111046 (PMC8583129; doi:10.3390/ijerph182111046)
Supplement: Supplementary file 1 [file ijerph-18-11046-s001.zip › ijerph-1379675-supplementary.pdf]

SUPPLEMENTAL INFORMATION

Table S1. Search terms

Exposure	Mechanism	Outcomes	Location
Wastewater	Irrigation	“Antimicrobial-resistan*”	Soil
Sewage	Agriculture	“Antibiotic-resistan*”	Field
Effluent		“Multidrug-resistan*”	“Surface water*”
“Reclaimed wastewater”			Groundwater
“Reclaimed water”			Aquifer
“Treated wastewater”			“Well water”
“Wastewater effluent”			Watershed
“Treated effluent”			Catchment
“Secondary-treated effluent”			“Water suppl*”
“Tertiary-treated effluent”			
“Sewage water”			
“Sewage effluent”			
Blackwater			
Greywater			

Text S1. Pubmed search string

((("greywater"[All Fields] OR "greywaters"[All Fields] OR ("blackwater"[All Fields] OR "blackwaters"[All Fields]) OR "Sewage effluent"[All Fields] OR "Sewage water"[All Fields] OR "Tertiary-treated effluent"[All Fields] OR "Secondary-treated effluent"[All Fields] OR "Treated effluent"[All Fields] OR "Wastewater effluent"[All Fields] OR "Treated wastewater"[All Fields] OR "Reclaimed water"[All Fields] OR "Reclaimed wastewater"[All Fields] OR ("effluent"[All Fields] OR "effluent s"[All Fields] OR "effluents"[All Fields]) OR ("sewage"[MeSH Terms] OR "sewage"[All Fields] OR "sewages"[All Fields]) OR ("waste water"[MeSH Terms] OR ("waste"[All Fields] AND "water"[All Fields]) OR "waste water"[All Fields] OR "wastewater"[All Fields] OR "wastewaters"[All Fields] OR "wastewater s"[All Fields])) AND ((Agriculture) OR (Irrigation))) AND ("antimicrobial resistan*"[All Fields] OR "antibiotic resistan*"[All Fields] OR "multidrug resistan*"[All Fields])) AND (((((((((Field) OR (Soil)) OR ("Agricultural field*")) OR ("Surface water*")) OR (Groundwater)) OR (Aquifer)) OR ("Well water")) OR (Watershed)) OR (Catchment)) OR ("Water suppl*"))

Table S2. PRISMA checklist

**Section/Topic**	**#**	**Checklist Item**	**Reported on page #**
**TITLE**			
Title	1	Identify the report as a systematic review, meta-analysis, or both.	Title
**ABSTRACT**			
Structured summary	2	Provide a structured summary including, as applicable: background; objectives; data sources; study eligibility criteria, participants, and interventions; study appraisal and synthesis methods; results; limitations; conclusions and implications of key findings; systematic review registration number.	Abstract
**INTRODUCTION**			
Rationale	3	Describe the rationale for the review in the context of what is already known.	Section 1, paragraph 3
Objectives	4	Provide an explicit statement of questions being addressed with reference to participants, interventions, comparisons, outcomes, and study design (PICOS).	Section 1, paragraph 3
**METHODS**			
Protocol and registration	5	Indicate if a review protocol exists, if and where it can be accessed (e.g., Web address), and, if available, provide registration information including registration number.	N/A
Eligibility criteria	6	Specify study characteristics (e.g., PICOS, length of follow-up) and report characteristics (e.g., years considered, language, publication status) used as criteria for eligibility, giving rationale.	Section 2.1, paragraph 2
Information sources	7	Describe all information sources (e.g., databases with dates of coverage, contact with study authors to identify additional studies) in the search and date last searched.	Section 2.1, paragraph 1
Search	8	Present full electronic search strategy for at least one database, including any limits used, such that it could be repeated.	SI Text S1
Study selection	9	State the process for selecting studies (i.e., screening, eligibility, included in systematic review, and, if applicable, included in the meta-analysis).	Section 2.1, paragraph 2

Data collection process	10	Describe method of data extraction from reports (e.g., piloted forms, independently, in duplicate) and any processes for obtaining and confirming data from investigators.	Section 2.2
Data items	11	List and define all variables for which data were sought (e.g., PICOS, funding sources) and any assumptions and simplifications made.	Section 2.2
Risk of bias in individual studies	12	Describe methods used for assessing risk of bias of individual studies (including specification of whether this was done at the study or outcome level), and how this information is to be used in any data synthesis.	N/A
Summary measures	13	State the principal summary measures (e.g., risk ratio, difference in means).	Section 2.2
Synthesis of results	14	Describe the methods of handling data and combining results of studies, if done, including measures of consistency (e.g., I2) for each meta-analysis.	Section 2.2
Risk of bias across studies	15	Specify any assessment of risk of bias that may affect the cumulative evidence (e.g., publication bias, selective reporting within studies).	N/A
Additional analyses	16	Describe methods of additional analyses (e.g., sensitivity or subgroup analyses, meta-regression), if done, indicating which were pre-specified.	N/A
**RESULTS**			
Study selection	17	Give numbers of studies screened, assessed for eligibility, and included in the review, with reasons for exclusions at each stage, ideally with a flow diagram.	Section 3, paragraph 1, Figure 1
Study characteristics	18	For each study, present characteristics for which data were extracted (e.g., study size, PICOS, follow-up period) and provide the citations.	Section 3 paragraph 1, section 3.1, paragraph 1, section 3.2 paragraph 1, Tables 1-2
Risk of bias within studies	19	Present data on risk of bias of each study and, if available, any outcome-level assessment (see Item 12).	N/A
Results of individual studies	20	For all outcomes considered (benefits or harms), present, for each study: (a) simple summary data for each intervention group and (b) effect estimates and confidence intervals, ideally with a forest plot.	Section 3.1, paragraphs 2-4, Section 3.2, paragraphs 2-7
Synthesis of results	21	Present results of each meta-analysis done, including confidence intervals and measures of consistency.	Section 3.1, paragraph 1,

			Section 3.2, paragraph 1
Risk of bias across studies	22	Present results of any assessment of risk of bias across studies (see Item 15).	N/A
Additional analysis	23	Give results of additional analyses, if done (e.g., sensitivity or subgroup analyses, meta-regression [see Item 16]).	N/A
**DISCUSSION**			
Summary of evidence	24	Summarize the main findings including the strength of evidence for each main outcome; consider their relevance to key groups (e.g., health care providers, users, and policy makers).	Section 4, paragraph 1
Limitations	25	Discuss limitations at study and outcome level (e.g., risk of bias), and at review level (e.g., incomplete retrieval of identified research, reporting bias).	Section 4, paragraphs 2-7
Conclusions	26	Provide a general interpretation of the results in the context of other evidence, and implications for future research.	Section 4, paragraphs 8-9, Section 5
**FUNDING**			
Funding	27	Describe sources of funding for the systematic review and other support (e.g., supply of data); role of funders for the systematic review.	N/A

Table S3. Characteristics of studies included in review

<table>
<tr><th>Author, year, site</th><th>WWI site, duration and comparison site</th><th>Soil sampling</th><th>Methods</th><th>AMR mechanism investigated</th><th>ARB/ARGs detected at WWI site vs. comparison site</th><th>Other relevant findings</th></tr>
<tr><td>Aleem et al. 2003

India</td><td>Field irrigated with untreated industrial wastewater mixed with domestic sewage

WWI duration:
10 years

Comparison site:
Field irrigated with groundwater</td><td>Composite samples from wheat rhizospheric soil</td><td>Free-living Azotobacter chroococcum isolated by culture

Antibiotic susceptibility testing by disc diffusion (36 isolates)</td><td>Resistance against:
Amoxycillin (25 µg)
Cloxacillin (30 µg)
Co-trimoxazole (30 µg)
Doxycycline (30 µg)
Methicillin (30 µg)
Nitrofurantion (30 µg)
Polymyxin-B (300 µg)
Rifampicin (2 µg)
Streptomycin (25 µg)
Sulphadiazine (300 µg)
Tetracycline (30 µg)</td><td>Findings reported for WWI site only

Almost all isolates resistant to multiple antibiotics

41% resistant to six antibiotics
92% resistant to nitrofurantion
86% resistant to polymyxin-B
81% resistant to co-trimoxazole</td><td>WWI site also had heavy metals</td></tr>
<tr><td>Ansari et al. 2007

India</td><td>Field irrigated with untreated industrial wastewater mixed with domestic sewage

WWI duration:
>20 years

Comparison site:
None</td><td>Composite samples at 15 cm depth at 4 locations per field

Alluvial, loamy soil</td><td>40 different bacteria isolated by culture

Antibiotic susceptibility testing by disc diffusion (40 isolates)

Plasmid-specific sequences identified by PCR and dot blot hybridization for resistant E. coli and Pseudomonas (12 isolates)</td><td>Resistance against:
Ampicillin (25 µg)
Chloramphenicol (25 µg)
Ciprofloxacin (30 µg)
Co-trimoxazole (25 µg)
Doxycycline (30 µg)
Gentamicin (30 µg)
Kanamycin (30 µg)
Nalidixic acid (30 µg)
Neomycin (30 µg)
Streptomycin (10 µg)
Tetracycline (30 µg)</td><td>Majority of isolates resistant to multiple antibiotics

75% resistant to tetracycline
58% resistant to doxycycline
50% resistant to ampicillin
50% resistant to nalidixic acid

IncP-specific plasmid sequences detected

IncQ, IncN, IncW and pMV158-type rolling circle plasmid-specific sequences not detected</td><td>WWI site also had heavy metals</td></tr>
<tr><td>Bahig et al. 2008

Egypt</td><td>Field irrigated with untreated wastewater

WWI duration:
Not reported

Comparison site:
Field irrigated with canal water</td><td>Samples at 0-5 cm depth (soil surface) and 20 cm depth (near plant roots)</td><td>Bacteria isolated by culture

Isolates screened for plasmids by lysis and gel electrophoresis (771 isolates)

Antibiotic susceptibility testing by disc diffusion for isolates that had plasmids (337 isolates)</td><td>Resistance against:
Ampicillin (100 µg)
Tetracycline (50 µg)
Kanamycin (10 µg)</td><td>Incidence of plasmids 25-50% higher in WWI site vs. comparison site

<table><tr><th></th><th>WWI</th><th>Control</th></tr><tr><td>Resistant to ampicillin</td><td>60%</td><td>63%</td></tr><tr><td>Resistant to tetracycline</td><td>25%</td><td>33%</td></tr><tr><td>Resistant to kanamycin</td><td>63%</td><td>53%</td></tr></table></td><td>Total culturable bacteria higher in WWI site

Metal resistance detected at both sites</td></tr>
<tr><td>Bougnom et al. 2019

Burkina Faso, Cameroon</td><td>Field irrigated with untreated domestic wastewater + hospital, agriculture, market and slaughterhouse waste

WWI duration:
20 years

Comparison site:
Non-irrigated field</td><td>Composite samples at 0-20 cm depth at 10 locations per field

WWI and comparison sites had similar soil properties (not specified)</td><td>High-throughput sequencing

ARGs identified from metagenome data</td><td>ARGs encoding:
Antibiotic inactivation enzymes, antibiotic target replacement, antibiotic target protection, efflux pumps</td><td>Number of ARGs for antibiotic inactivation enzyme genes 6% lower in comparison site

Number of ARGs for other resistance mechanisms 2% higher in WWI site

<table><tr><th></th><th>WWI</th><th>Control</th></tr><tr><td>Inactivation enzyme</td><td>65%</td><td>72%</td></tr><tr><td>Target replacement</td><td>15%</td><td>13%</td></tr><tr><td>Target protection</td><td>12%</td><td>9%</td></tr><tr><td>Efflux pump</td><td>6%</td><td>9%</td></tr></table></td><td>--</td></tr>
</table>

<table>
<tr><th>Author, year, site</th><th>WWI site, duration and comparison site</th><th>Soil sampling</th><th>Methods</th><th>AMR mechanism investigated</th><th>ARB/ARGs detected at WWI site vs. comparison site</th><th>Other relevant findings</th></tr>
<tr><td>Bougnom et al. 2020

Burkina Faso, Cameroon</td><td>Same as above</td><td>Composite samples at 0-20 cm depth at 10 locations per field, 6 fields per city, 3 cities</td><td>Same as above</td><td>Non-targeted ARGs and *Enterobacteriaceae* plasmid replicons</td><td>Transferable ARGs made up 33/45 of ARGs at WWI site and 26/39 at comparison site

12 ARG types only detected in WWI site (5 only in comparison site), 2 plasmid replicon groups only detected in WWI site (3 only in comparison site)

27% more transferable ARGs in WWI site</td><td>Enrofloxacin, oxytetracycline, sulfamethoxazole found at higher concentration in WWI site</td></tr>
<tr><td>Broszat et al. 2014

Mexico</td><td>Field irrigated with untreated municipal wastewater

WWI duration:
8, 10, 85, and 100 years

Comparison site:
Rain-fed field</td><td>Composite samples at 0-30 cm depth at 48 locations per field</td><td>Bacteria isolated by culture

Antibiotic susceptibility testing by disc diffusion (192 isolates)

ARGs detected by PCR among isolates resistant to ciprofloxacin and sulfamethoxazole</td><td>Resistance against:
Ampicillin (25 µg)
Chloramphenicol (30 µg)
Erythromycin (10 µg)
Gentamicin (10 µg)
Kanamycin (30 µg)
Oxacillin (5 µg)
Streptomycin (25 µg)
Ciprofloxacin (5 & 10 µg)
Doxycycline (30 µg)
Tetracycline (30 µg)
Vancomycin (30 µg)
Sulfamethoxazole (25 µg)

ARGs:
Sulfonamide (*sul*) and fluoroquinolone (*qnr*) resistance genes</td><td><table>
<tr><th></th><th>WWI</th><th>Control</th></tr>
<tr><td>Resistant to ciprofloxacin</td><td>3%</td><td>0%</td></tr>
<tr><td>Resistant to sulfamethoxazole</td><td>33%</td><td>18%</td></tr>
<tr><td>Resistant to ≥1 antibiotic</td><td>51%</td><td>34%</td></tr>
<tr><td>Resistant to ≥2 antibiotics</td><td>25%</td><td>6%</td></tr>
<tr><td>Resistant to 3 antibiotics</td><td>3%</td><td>2%</td></tr>
<tr><td>Resistant to >3 antibiotics</td><td>9%</td><td>0%</td></tr>
</table>
sul and *qnr* genes detected in total DNA from soil samples but not in DNA from isolates</td><td>Total organic content higher in WWI site</td></tr>
<tr><td>Cerqueria et al. 2018

Spain</td><td>Field irrigated with water from channel with up to 92% treated effluent from 10 treatment plants

WWI duration:
Not reported

Comparison site:
Field irrigated with ground- and/or rainwater</td><td>Samples from 4 locations per field

Sandy loam</td><td>ARGs and mobile genetic elements quantified by qPCR with DNA extracted from soil</td><td>ARGs:
sul1
*bla*TEM
*bla*OXA-58
*bla*CTX-M-32
*mec*A
qnrS1
tetM

Mobile genetic elements:
intI1</td><td>Relative abundance of tetM, mecA, qnrS1 and *bla*OXA-58 genes higher in WWI site

Relative abundance of *bla*CTX-M-32 genes higher in comparison site</td><td>ARGs and *intI1* genes found in leaves/fruits at 100-10 times lower abundance than in roots/soil</td></tr>
<tr><td>Cerqueria et al. 2019

Spain</td><td>(1) Field irrigated with water from channel with up to 92% treated effluent from 10 treatment plants

(2) Field irrigated with water from river that has <18% treated effluent

WWI duration:
Not reported

Comparison site:
Field irrigated with groundwater</td><td>Samples from 5 locations per field</td><td>ARGs and mobile genetic elements quantified by qPCR with DNA extracted from soil</td><td>ARGs:
sul1
*bla*TEM
*bla*OXA-58
*bla*CTX-M-32
*mec*A
qnrS1
tetM

Mobile genetic elements:
intI1</td><td>*bla*TEM detected in all samples, other ARGs ranged from 10-73% in prevalence

Absolute abundance of *bla*TEM genes highest in site irrigated with river water containing <18% treated wastewater

Absolute abundance of *qnrS1* genes higher in both WWI sites

Absolute abundance of *intI1* genes higher in comparison site</td><td>*bla*TEM had highest abundance of all ARGs, other ARGs at low levels or below level of detection</td></tr>
</table>

Author, year, site	WWI site, duration and comparison site	Soil sampling	Methods	AMR mechanism investigated	ARB/ARGs detected at WWI site vs. comparison site	Other relevant findings
Chen et al. 2014<br><br>China	(1) Field irrigated with treated wastewater directly or from rivers that receive effluent<br><br>(2) Field irrigated with untreated wastewater until 6-7 years ago, irrigated with ground- and/or rainwater since<br><br><u>WWI duration</u>:<br>Not reported<br><br><u>Comparison site</u>:<br>Non-irrigated field	Composite samples at 0-5 cm depth at 10 locations per field	ARB enumerated on agar plates containing antibiotics, normalized by the total number of cultivatable bacteria<br><br>ARGs quantified by qPCR with DNA extracted from soil, normalized by 16S rRNA genes	<u>Resistance against</u>:<br>Oxytetracycline (20 mg/L)<br>Tetracycline (20 mg/L)<br>Sulfadiazine (50 mg/L)<br>Sulfamethoxazole (50 mg/L)<br><br><u>ARGs</u>:<br>13 tetracycline resistance genes (*tetA, tetB, tetC, tetD, tetE, tetG, tetK, tetL, tetM, tetO, tetS, tetQ, tetX*)<br><br>3 sulfonamide resistance genes (*sul1, sul2, sul3)*	Relative abundance of sulfadiazine-resistant bacteria highest in previous WWI site, no other differences between sites in relative abundance of ARBs<br><br>Relative abundance of *tetA*, *tetC*, *tetE*, *tetG*, *tetS*, *sul1*, *sul3* higher in current and previous WWI sites, no other differences between sites in relative abundance of ARGs<br><br>Sum of relative abundances of *tet* and *sul* genes higher in current and previous WWI sites<br><br>No difference in relative abundance of ARGs between current and previous WWI sites	Antibiotics least frequently detected in comparison site, highest concentration in current and previous WWI sites<br><br>ARB abundance not correlated with antibiotic concentrations<br><br>Sum of *tet* genes and sum of *sul* genes correlated with OTC concentration<br><br>*sul* genes correlated with SDZ & SMX concentration
Chigor et al. 2020<br><br>Nigeria	Earthen pots irrigated with secondary treated wastewater<br><br><u>WWI duration</u>:<br>Practiced in the area for >30 years, earthen pots irrigated for 6 weeks<br><br><u>Comparison site</u>:<br>None	Composite samples at 0–20 cm depth from earthen pots	*E. coli* isolated by culture and confirmed by PCR<br><br>Antibiotic susceptibility testing by disc diffusion for isolates confirmed as *E. coli* by PCR (4 isolates)	<u>Resistance against</u>:<br>Amoxicillin (10 µg)<br>Ampicillin (5 µg)<br>Penicillin (10 µg)<br>Cloxacillin (5 µg)<br>Cefuroxime (30 µg)<br>Streptomycin (10 µg)<br>Rifampicin (5 µg)<br>Metronidazole (50 µg),<br>Sulfamethoxazole (25 µg)<br>Trimethoprim (5 µg)<br>Vancomycin (30 µg)<br>Erythromycin (15 µg)<br>Clarithromycin (15 µg)<br>Chloramphenicol (30 µg)<br>Ciprofloxacin (5 µg)<br>Norfloxacin (10 µg)<br>Tetracycline (30 µg)<br>Imipenem (10 µg)	100% resistant to ≥5 antibiotics<br><br>100% resistant to amoxicillin, ampicillin, penicillin, cloxacillin, rifampicin, metronidazole, sulfamethoxazole, trimethoprim, vancomycin, erythromycin, clarithromycin, tetracycline<br><br>75% resistant to ciprofloxacin and norfloxacin	1 out of 4 soil isolates had *lt* gene encoding enterotoxigenic *E. coli*
Dalkmann et al. 2012<br><br>Mexico	Field irrigated with untreated municipal wastewater<br><br><u>WWI duration</u>:<br>1.5, 3, 6, 8, 85, and 100 years<br><br><u>Comparison site</u>:<br>Rain-fed field	Composite samples at 0-30 cm depth with 12 subsamples per location and 4 locations per field<br><br>Soil classified as Leptosols, Vertisols, and Phaeozems	ARGs quantified by qPCR with DNA extracted from soil, normalized by 16S rRNA genes	<u>ARGs</u>:<br>2 sulfonamide resistance genes (*sul1*, *sul2*)<br><br>3 fluoroquinolone resistance genes (*qnrA*, *qnrB*, *qnrS*)	*sul1* and *sul2* detected in both sites<br><br>Absolute abundance 150-1500 times higher for *sul1* and 50-520 times higher for *sul2* in WWI site<br><br>Relative abundance 100 times higher for *sul1* and 20 times higher for *sul2* in WWI site<br><br>Absolute abundance of *sul1 and sul2* genes increased with increasing years of irrigation, relative abundance did not<br><br>No *qnrA* genes detected<br><br>*qnrB* and *qnrS* genes detected in two WWI sites (irrigated for 6 and 100 years)	Ciprofloxacin, sulfamethoxazole, carbamazepine concentration in soil increased asymptotically with years of irrigation, steady state at ~25 years<br><br>Antibiotic accumulation was dependent on soil type and acidity of antibiotic

**Author, year, site**	**WWI site, duration and comparison site**	**Soil sampling**	**Methods**	**AMR mechanism investigated**	**ARB/ARGs detected at WWI site vs. comparison site**	**Other relevant findings**
Han et al. 2016<br><br>Australia	Urban park irrigated with tertiary treated wastewater<br><br>Duration of WWI:<br>Not reported<br><br>Comparison site:<br>(1) Urban park irrigated with potable water<br><br>(2) Pristine soil from remote national parks	3 replicate samples at 10 cm depth per park, each sample consists of 5 sub-samples within 50 m^2<br><br>Loamy sand	ARGs and mobile genetic elements quantified by qPCR with DNA extracted from soil	84 ARGs encoding resistance to aminoglycosides, Classes A, B, C and D beta-lactam, erythromycin, quinolones and fluoroquinolones, macrolide lincosamide streptogramin_b (MLS_b), multidrug, tetracycline, vancomycin<br><br>Mobile genetic elements:<br>*intI1*<br>*tnpA* gene of IS6 family transposons	ARG detected at all sites, total of 40 unique ARGs<br><br>Number of different ARGs higher at WWI site than both comparison sites, no difference between two comparison sites<br><br>Abundance of ARGs 815-4300 times higher at WWI site than national parks<br><br>Abundance of ARGs 150-1240 times higher at urban park without WWI than national parks<br><br>No difference in relative abundance of *intI1* and *tnpA* genes between sites	Genes encoding resistance to classes A, B, C, and D beta-lactam were most frequently detected<br><br>Abundance of ARGs correlated positively with soil pH and negatively with total nitrogen content<br><br>WWI site had different bacterial community structure than comparison sites
Jechalke et al. 2015<br><br>Mexico	Field irrigated with untreated municipal wastewater (65% domestic sewage, 20% service sector waste, 15% industrial waste)<br><br>Duration of WWI:<br>1.5, 3, 6, 8, 85, and 100 years, irrigated 10-12 times per year for 6-24 hours per irrigation event<br><br>Comparison site:<br>Rain-fed field	Composite samples at 0-30 cm depth with 12 subsamples per location and 4 locations per field<br><br>Sampled in dry season only<br><br>Soil classified as Leptosols, Vertisols, and Phaeozem	ARGs and mobile genetic elements quantified by qPCR with DNA extracted from soil, normalized by 16S rRNA genes	ARGs:<br>*tetW*<br>*tetQ*<br>*aadA*<br>*qacE+qacED1*<br><br>Mobile genetic elements:<br>*intI1*<br>IncP-1plasmids (*korB*)	Target genes below the limit of detection in comparison site<br><br>Absolute abundance of all target genes other than tetQ increased with increasing years of irrigation<br><br>Relative abundance of tetQ decreased with increasing years of irrigation, for other target genes there was no correlation with duration of irrigation	Concentrations of Zn, Cu, Pb, Ni, Cr, P, and S increased with years of irrigation<br><br>Absolute abundance of all target genes other than tetQ positively correlated with total organic content<br><br>Absolute abundance of tetQ positively correlated with electrical conductivity<br><br>Gene abundance not correlated with soil pH
Kampouris et al. 2020<br><br>Germany	Field irrigated with secondary treated wastewater, sometimes mixed with digested sludge<br><br>Duration of irrigation:<br>50 years<br><br>Comparison site:<br>Period of irrigation compared to period without irrigation<br><br>Also, lab experiment where soils were irrigated with treated wastewater and freshwater	Soil pore water sampled at 40, 80, 120 cm depth<br><br>Soil classified as sandy (cambisol)<br><br>Samples over 1 year, during low-, medium- and high-intensity irrigation and after a 4-month irrigation break<br><br>12 forest soil samples at 60 cm depth for lab experiment	ARGs and mobile genetic elements quantified by qPCR from DNA directly extracted from water and soil, normalized by 16S rRNA genes	ARGs:<br>*sul1*<br>*tetM*<br>*qnrS*<br>$bla_{OXA\text{-}58}$<br>$bla_{CTX\text{-}M\text{-}32}$<br>$bla_{TEM}$<br><br>Mobile genetic elements:<br>*intI1*	All target genes detected in subsoil pore water in WWI site<br><br>Relative abundance of all target genes 0.5-2 orders of magnitude higher during high-intensity irrigation compared to after irrigation break<br><br>Relative abundance of *sul1*, *intI1*, *qnrS*, and $bla_{OXA\text{-}58}$ positively correlated with irrigation intensity<br><br>In lab experiment, relative abundance of *sul1*, *intI1*, *qnrS*, *tetM* and $bla_{OXA\text{-}58}$ increased concurrently with irrigation with treated wastewater	Relative abundance of *sul1* and *qnrS* correlated with temperature<br><br>Relative abundance of *sul1* correlated with precipitation<br><br>Humidity not correlated with any target genes<br><br>Effects observed at all three depths

<table>
<tr><th>Author, year, site</th><th>WWI site, duration and comparison site</th><th>Soil sampling</th><th>Methods</th><th>AMR mechanism investigated</th><th>ARB/ARGs detected at WWI site vs. comparison site</th><th>Other relevant findings</th></tr>
<tr><td>Luneberg et al. 2017

Mexico</td><td>Field irrigated with untreated wastewater

Duration of irrigation:
>80 years

Comparison site:
Rain-fed field</td><td>4 soil cores per field at 0-20 cm
Soil classified as haplic Phaeozem
Soil cores stained with dye to see flow paths, then irrigated with untreated wastewater with and without sulfamethoxazole and ciprofloxacin</td><td>ARGs quantified by qPCR from DNA extracted from soil, normalized by 16S rRNA genes</td><td>ARGs:
sul1
sul2
qnrB
qnrS</td><td>*sul1* detected in comparison site

Relative abundance of *sul1* in soil core from comparison site increased by up to 3 orders of magnitude after irrigation with spiked wastewater

Relative abundance of *sul1* in soil core from WWI site increased by <1 order of magnitude after irrigation experiment

sul2 not detected in comparison site but became detectable in stained soil (along flow path) after irrigation experiment

qnrB and *qnrS* not detected</td><td>Ciprofloxacin only accumulated along flow path after irrigation experiment

Sulfamethoxazole increased in both stained and unstained soil but was higher along the flow path

Relative abundance of *sul1* and *sul2* higher in stained soil (along flow path) than unstained soil</td></tr>
<tr><td>Malik and Aleem 2011

India</td><td>Field irrigated with untreated industrial wastewater mixed with domestic sewage

WWI duration:
10 years

Comparison site:
Field irrigated with groundwater</td><td>Composite samples at 15 cm depth</td><td>*Pseudomonas spp.* isolated by culture

Antibiotic susceptibility testing by disc diffusion (96 isolates)

Several multi-resistant isolates screened for plasmids by gel electrophoresis</td><td>Resistance against:
Amoxycillin (25 µg)
Ampicillin (30 µg)
Chloramphenicol (30 µg)
Ciprofloxacin (30 µg)
Cloxacillin (30 µg)
Cotrimoxazole (30 µg)
Doxycycline (30 µg)
Erythromycin (15 µg)
Gentamicin (30 µg)
Kanamycin (30 µg)
Methicillin (30 µg)
Nalidixic acid (30 µg)
Neomycin (30 µg)
Nitrofurantoin (30 µg)
Polymyxin-B (300 µg)
Rifampicin (2 µg)
Streptomycin (25 µg)
Sulphadiazine (300 µg)
Tetracycline (30 µg)</td><td><table><tr><th></th><th>WWI</th><th>Control</th></tr><tr><td>Resistant to sulphadiazine</td><td>88%</td><td>27%</td></tr><tr><td>Resistant ampicillin</td><td>79%</td><td>73%</td></tr><tr><td>Resistant to erythromycin</td><td>79%</td><td>21%</td></tr></table>
In WWI site, 15% of isolates resistant to 14 antibiotics, 19% resistant to 13 antibiotics, 15% resistant to 11 antibiotics

In comparison site, 29% of isolates resistant to 8 antibiotics</td><td>WWI site also had heavy metals</td></tr>
<tr><td>Marano et al. 2019

Israel</td><td>Fields irrigated with secondary and tertiary treated wastewater
WWI duration:
Not reported
Comparison site:
Field irrigated with surface, ground- or desalinated water
Also, experimental orchard and lysimeters irrigated with tertiary treated wastewater vs. freshwater</td><td>Samples at 10-15 cm depth
Soil in fields classified as clay, sandy loam and sandy clay
Experimental orchard had clayey soil
Lysimeters filled with sandy clay, sandy loamy and loamy sandy soil</td><td>ARGs and mobile genetic elements quantified by qPCR from DNA extracted from soil, normalized by 16S rRNA genes</td><td>ARGs:
bla_{GES}
bla_{OXA2}
bla_{OXA10}
bla_{TEM}
$bla_{CTX\text{-}M\text{-}32}$
qnrS

Mobile genetic elements:
intI1</td><td>*intI1* was quantifiable in most soil samples

Both absolute and relative abundance of intI1 for all soil types was higher in lysimeter samples irrigated with treated wastewater vs. freshwater

Abundance of *intI1* in commercial fields positively correlated with *intI1* in wastewater effluent for sandy soils but not clayey soils

Most ARGs were below the level of quantification in soil samples and not correlated with ARG abundance in wastewater effluent</td><td>--</td></tr>
</table>

<table>
<tr><th>Author, year, site</th><th>WWI site, duration and comparison site</th><th>Soil sampling</th><th>Methods</th><th>AMR mechanism investigated</th><th>ARB/ARGs detected at WWI site vs. comparison site</th><th>Other relevant findings</th></tr>
<tr><td>McLain and Williams 2010

USA</td><td>Soil from water storage basin recharged with tertiary treated wastewater

<u>WWI duration</u>:
>20 years

<u>Comparison site</u>:
Soil from water storage basin recharged with groundwater</td><td>Soil cores at 0-5, 10-15, 25-50 cm depth</td><td>Enterococcus isolated by culture

Antibiotic susceptibility by broth microdilution (61 isolates)</td><td><u>Resistance against:</u>
Tigecycline
Tetracycline
Chloramphenicol
Daptomycin
Streptomycin
Tylosin tartrate
Quinupristin/dalfopristin
Linezolid
Nitrofurantoin
Penicillin
Kanamycin
Erithromycin
Ciprofloxacin
Vancomycin
Lincomycin
Gentamicin</td><td><table><tr><td></td><td>WWI</td><td>Control</td></tr><tr><td>Susceptible to all antibiotic</td><td>36%</td><td>7%</td></tr><tr><td>Resistant to 4-6 antibiotics</td><td>9%</td><td>25%</td></tr></table>
>50% of isolates resistant to daptomycin and lincomycin in both sites

More isolates resistant to quinupristin/dalfopristin (27% vs. 4%) and kanamycin (12% vs. 0%) in WWI site

More isolates resistant to erythromycin (43% vs. 21%), tetracycline (21% vs. 0%), ciprofloxacin (57% vs. 24%) and tylosin tartrate (25% vs. 3%) in comparison site</td><td>--</td></tr>
<tr><td>Negreanu et al. 2012

Israel</td><td>Fields irrigated with secondary treated wastewater

<u>WWI duration</u>:
6, 12, 15 years

<u>Comparison site</u>:
Field irrigated with freshwater, including aquifer recharged with secondary treated wastewater</td><td>Samples at 5 cm depth from 4 different areas

Soils classified as vertisol (52-60% clay), loam (20% clay) and dune quartz sand</td><td>Relative abundance of resistant bacteria enumerated by serial dilution, by comparing colony forming units (CFUs) on plates with vs. without antibiotics

ARGs quantified by qPCR from DNA extracted from soil, normalized by 16S rRNA genes</td><td><u>Resistance against</u>:
Tetracycline (20 mg/L)
Ciprofloxacin (4 mg/L)
Erythromycin (10 mg/L)

<u>ARGs</u>:
sul1
sul2
ermB
ermF
tetO
qnrA</td><td>Relative abundance of resistant bacteria similar or higher in comparison site than WWI site

Absolute abundance of ARGs similar or higher in comparison site than WWI site in 3 out of 4 areas, higher in WWI site than comparison site in 1 area

Relative abundance of ARGs higher in comparison site than WWI site in 3 out of 4 areas, higher in WWI site than comparison site in 1 area</td><td>Tetracycline and ciprofloxacin resistant bacteria and ARGs detected in secondary treated wastewater

Tetracycline and ciprofloxacin resistance positively correlated with soil moisture</td></tr>
<tr><td>Palacios et al. 2017a

Mexico</td><td>(1) Field irrigated with water from river that receives untreated wastewater

(2) Field irrigated with untreated wastewater from river until >10 years ago

<u>WWI duration</u>:
Not reported

<u>Comparison site</u>:
Rain-fed field</td><td>Samples at 0-15, 15-30 and 30-50 cm depth

Soil classified as Orthid Aridisol with well-developed pedogenic horizons</td><td>Ampicillin-resistant bacteria isolated by plating on growth media with ampicillin

Antibiotic susceptibility by Bauer-Kirby method for ampicillin-resistant isolates (111 isolates)</td><td><u>Resistance against:</u>
Ampicillin (250 mg/L)

24 antibiotics (6 for Gram-negative bacteria, 6 for Gram-positive bacteria, 12 for both)</td><td>Prevalence of multi-resistant bacteria higher at current WWI site than previous WWI site, which was higher than comparison site

Prevalence of multi-resistant bacteria only different for Gram-positive bacteria between previous WWI site and comparison site

Higher number of isolates from current WWI site resistant to ≥6 antibiotics</td><td>Prevalence of multi-resistant bacteria not affected by soil depth</td></tr>
<tr><td>Palacios et al. 2017b

Mexico</td><td>Recreational parks irrigated with tertiary treated wastewater

<u>WWI duration</u>:
Not reported

<u>Comparison site</u>:
Distance from WWTP</td><td>Samples at 0-10 cm depth from 28 parks

Soil classified as silty clay</td><td>Bacteria concentrated by membrane filtration

Antibiotic susceptibility by plating with and without antibiotics</td><td><u>Resistance against:</u>
Ampicillin (10 μg)
Riphampicin (5 μg)
Chloramphenicol (30 μg)
Ciprofloxacin (5 μg)
Gentamicin (10 μg)
Trimethoprim-sulphametoxazole (300 μg)</td><td>392 multi-resistant isolates identified

166 isolates resistant to ampicillin
171 isolates resistant to gentamicin

Higher number of multi-resistant bacteria closer (<6 km) to the wastewater plant</td><td>Number of multi-resistant bacteria lower during the dry season</td></tr>
</table>

Author, year, site	WWI site, duration and comparison site	Soil sampling	Methods	AMR mechanism investigated	ARB/ARGs detected at WWI site vs. comparison site	Other relevant findings
Pan and Chu 2018<br><br>China	(1) Fields irrigated with untreated domestic wastewater<br><br>(2) Fields irrigated with fishpond water<br><br><u>WWI duration</u>:<br>>20 years<br><br><u>Comparison site</u>:<br>Field with no cultivation	Samples at 0-10 cm and 10-20 cm depth from 5 random 100 m x 100m plots per site	ARGs quantified by qPCR from DNA directly extracted from soil, normalized by 16S rRNA genes	<u>ARGs:</u><br>Tetracycline resistance genes (*tetA, tetB, tetC, tet E, tetM, tetO, tet S, tetX*)<br><br>Sulfonamide resistance genes (*sul1, sul2, sul3*)	No ARGs detected at comparison site<br><br>Relative abundance of ARGs higher in field irrigated with fishpond water than in WWI site.<br><br>Almost all *tet* genes positively correlated with tetracycline concentration in soil<br><br>All *sul* genes and *tetE* positively correlated with sulfamethazine concentration in soil	No antibiotics detected at comparison site<br><br>Fishpond water had higher concentration of both antibiotics than domestic wastewater<br><br>Tetracycline concentration higher at 0-10 cm than 10-20 cm<br><br>Sulfamethazine concentration similar at different depths<br><br>Relative abundance of ARGs not significantly different between two soil depths
Shafiani and Malik 2013<br><br>India	Field irrigated with untreated industrial wastewater mixed with domestic sewage<br><br><u>WWI duration</u>:<br>10 years<br><br><u>Comparison site</u>:<br>None	Composite samples at 0-15 cm depth	*Pseudomonas spp.* isolated by culture<br><br>Antibiotic susceptibility testing by disc diffusion (40 isolates)	<u>Resistance against</u>:<br>Amoxycillin (25 µg)<br>Chloramphenicol (30 µg)<br>Cloxacillin (30 µg)<br>Doxycycline (30 µg)<br>Methicillin (30 µg)<br>Nalidixic acid (30 µg)<br>Tetracycline (30 µg)	100% resistant to cloxacillin<br>58% resistant to methicillin<br>25% resistant to 4 antibiotics	Pesticides also present in WWI site
Troiano et al. 2018<br><br>Israel	Field irrigated with greywater treated by recirculating vertical flow constructed wetland<br><br><u>WWI duration</u>:<br>>7 years<br><br><u>Comparison site</u>:<br>Field irrigated with freshwater	Duplicate samples at 5 cm depth at three different greywater treatment systems	Relative abundance of resistant by comparing colony forming units (CFUs) on plates with and without tetracycline<br><br>Tetracycline resistant bacteria isolated by culture<br><br>Tetracycline-resistant strains (n=24) tested by culture for resistance against 3 additional antibiotics<br><br>Antibiotic susceptibility by broth microdilution<br><br>Identification by 16s rRNA gene sequencing<br><br>ARGs detected by PCR among tetracycline-resistant strains	<u>Resistance against</u>:<br>Tetracycline (20 mg/L)<br>Amoxicillin (20 mg/L)<br>Ciprofloxacin (20 mg/L)<br>Kanamycin (20 mg/L)<br><br><u>ARGs</u>:<br>Beta-lactamase genes (*bla*$_{TEM}$, *bla*$_{CTXM-32}$, *bla*$_{SHV}$, *bla*$_{OXA-2}$, *bla*$_{OXA-10}$)<br><br>Tetracycline resistance genes (*tet39*, *tetA*, *tetB tetM*, *tetQ*, *tetW*)	Isolated ARB were not obligatory pathogens<br><br>Tetracycline-resistant bacteria detected in soil in two out of three greywater treatment systems<br><br>No difference in tetracycline resistant bacteria between WWI and comparison sites	Only *tet39* detected among tetracycline-resistant bacteria<br><br>All isolates positive for *tet39* were also positive for at least one beta-lactamase gene

Author, year, site	WWI site, duration and comparison site	Soil sampling	Methods	AMR mechanism investigated	ARB/ARGs detected at WWI site vs. comparison site	Other relevant findings
Wang et al. 2014a<br><br>China	Public parks irrigated with treated wastewater<br><br>WWI duration:<br>Not reported<br><br>Comparison site:<br>Pristine remote parks	Samples from rhizosphere soil, non-rhizosphere soil and wetland soil	ARGs detected by PCR from DNA directly extracted from soil<br><br>Commonly detected ARGs quantified by qPCR, normalized by 16S rRNA genes	ARGs:<br>15 tetracycline genes<br>4 beta-lactamase genes<br>3 quinolone genes<br><br>Mobile genetic elements:<br>*intl1*	*tetG*, *tetW*, *sul1*, *sul2* and *intl1* detected in all WWI sites<br><br>*sul1* and *intl1* detected in comparison site<br><br>Abundance and diversity of ARGs higher in WWI site than comparison site<br><br>Absolute abundance of sul1 genes: 1.69 x 10^8 copies per g dry soil at WWI site, 9.08 x 10^7 copies per g dry soil at comparison site<br><br>Absolute abundance of intl1 genes: 7.62 x 10^7 copies per g dry soil at WWI site, 2.61 x 10^7 copies per g dry soil at comparison site	Antibiotics detected in WWI site but not comparison site<br><br>ARG abundance did not differ between rhizosphere, non-rhizosphere and wetland samples<br><br>Soil pH (all >7.0) negatively correlated with ARG abundance<br><br>Carbon, nitrogen and organic matter content positively correlated with ARG abundance
Wang et al. 2014b<br><br>China	Urban parks irrigated with treated wastewater in seven cities<br><br>WWI duration:<br>3-12 years<br><br>Comparison site:<br>Urban parks not irrigated with reclaimed water in same cities	Samples at 0-10 cm depth from four plots per site	High-throughput qPCR from DNA directly extracted from soil	ARGs:<br>285 different ARGs<br><br>Mobile genetic elements:<br>9 transposase genes	Average of 87 ARGs detected in WWI sites, 81 ARGs detected in comparison sites<br><br>Among 147 unique ARGs detected, 105 were enriched in WWI sites<br><br>ARG abundance 99-8655 times higher in WWI sites than comparison sites<br><br>Abundance of transposase genes up to 2959 times higher in WWI sites than comparison sites	Antibiotic concentrations higher in WWI sites
